# Supplementary material for: ‘The world is just so fast, and I’m not fast… it’s just really, really difficult to keep up’: A qualitative exploration of the lived experience of adults with Developmental Coordination Disorder
Source: PLoS One. 2026 May 28;21(5):e0350273. doi: 10.1371/journal.pone.0350273 (PMC13218497; doi:10.1371/journal.pone.0350273)
Supplement: Attachment — Submitted filename: Response to reviewers.docx [file pone.0350273.s004.docx]

**Reviewer 1**

This paper qualitatively investigates the lived experience of adults with DCD, an important and underexplored topic. The study addresses a gap in the literature, and several aspects—such as the reflexive approach and consideration for participant comfort—are commendable. However, there are areas that require further development, particularly in the Results section, before the manuscript is ready for publication. Below, I outline my general comments and specific suggestions for improvement.
We thank the reviewer for their kind words. We really appreciate the time they have taken to review the manuscript. We have worked through all of their suggestions and believe the manuscript is much stronger for their input.

General comments
Throughout the paper, I recommend using the term “condition” instead of “disorder” (except when referring to DCD by its full name, as this is the official diagnostic term). Similarly, “co-occurrence” is preferable to “co-morbidity.”
We agree this terminology is preferable and thus have changed this throughout the manuscript.

The manuscript notes that participants were recruited cross-culturally, which is a strength, but this perspective is not fully developed in the aims, research questions, results, or discussion. Please expand on why cultural differences might matter for DCD experiences and reflect these differences in the analysis.
The rationale for taking a lifespan, cross-cultural qualitative approach is mentioned but not sufficiently unpacked. There’s no elaboration on why cross-cultural recruitment might reveal different experiences, or how qualitative interviews address the identified research gap better than other methods.
Although we advertised the study to all English speaking countries, we were only able to recruit one individual from overseas (New Zealand). This participant grew up in the UK - we have not mentioned this as they wanted this to be redacted to ensure anonymity. They were therefore reflecting on experiences within the UK (childhood) and New Zealand (adulthood). One international participant was not enough to explore cross cultural differences, so we have removed all mention of this from the manuscript. We have added some blurb about this to the limitation section.

The phrase “formally known as Dyspraxia” could be misleading. Dyspraxia is still widely used in educational, healthcare, and employment contexts, whereas DCD is primarily used in research and has formal diagnostic criteria (unlike Dyspraxia). Please clarify the diagnostic terminology to avoid confusion.
We have amended the transcript to reflect that DCD is the diagnostic term, however it is also known as and referred to as Dyspraxia.

Abstract
Include the mean age and SD of participants
This information was not collected from participants. We appreciate this information may have been helpful in determining generational changes in experience – so we have included this as a limitation in the discussion.

Intro
As DCD is primarily a movement condition, please introduce this before discussing sensory integration difficulties, aligning the description with the four DSM-5 diagnostic criteria.
We have removed the sensorimotor integration aspect to remove confusion. We have instead listed the diagnostic criteria from the DSM-V.

Where possible, cite original research evidence for the persistence of DCD into adulthood, rather than secondary recommendations seen in the EADC document.
We have included a sentence about this being reflected in academic literature too. Further discussion of this literature is also later on in the introduction.

At times, the literature is presented as a list of findings without deeper synthesis. Please use the literature to build an argument that logically narrows toward the research gap. Here, findings are introduced quickly and sequentially without enough critical commentary to connect them.
We have endeavoured to restructure the introduction to allow for greater synthesis and direction towards the research gap.

Add the research questions in line with APA recommendations for qualitative research.
We have aligned the paper to the COREQ checklist for qualitative research as per PLOS recommendations. The checklist for this can now be found in supplementary material 1.

Clarify abstract concepts (e.g., “moving through space,” “illusive resolution”) with more concrete language.
We have changed moving through space to *“being able to successfully navigate their environment”*, and illusive resolution to *“which were not easily overcome”*. We hope this clarifies the intended meaning within the manuscript.

Acknowledge that DCD can be diagnosed at any age.
We agree this was unclear in the manuscript. We have added a caveat to the diagnosis section which states *“DCD is often, although not exclusively diagnosed in childhood”*.

Where statements imply direct causation (e.g., challenges directly linked to poorer well-being), please expand and clarify.
We have amended the language in both the results and discussion to mitigate for implied causality. Rather the changes allude to relationships shown in the literature.

The sentence “Although many of these studies have focused on university populations” should be supported with a reference.
We have now included references to justify the inclusion of this sentence.

Methods
As there were only 7 questions used in this study, I recommend including key questions (or all of them) in the main script to make the study’s focus clear to readers.
We have now included these as a table (with prompts) in the main manuscript (Table 1). We agree this will facilitate easier deciphering of findings.

Provide more detail about the diagnostic evidence you used for the participants, what age were they diagnosed, who by and in which country.
We did not ask for evidence of a formal diagnosis to be provided for this study, as for many, DCD is diagnosed in childhood. As we were interviewing adults, there were concerns that participants may not be able to access formal evidence of a diagnosis. We have however acknowledged this as a limitation in the discussion section.

Include justification for sample size with reference to previous research.
We have justified the sample size in the participants section of the methods, through both previous literature, and our experience of data saturation.

Age, gender, and country breakdown are provided, but not linked to the research aims.
Participant demographics were provided to give insight into the participants that were recruited, rather than these being linked directly to research aims (other than age – for which we were looking specifically at the adult population). As such, we have referenced these demographics in the discussion but not specifically analysed the data on this basis. Particularly as this would not be possible for many of these demographics (e.g. sex where there were only two males, and country, where only one was international, and this participant grew up in the UK).

It is a strength that interview questions were sent to some participants in advance and that member checking was used—this demonstrates a participant-centred, reflexive approach.
We thank the reviewer for their kind words, we wanted to make sure all participants felt their stories were a true reflection of their experiences, and we were grateful for their input through member checking.

Results
This section contains valuable, vivid participant accounts, but requires reorganisation for clarity and alignment with APA style. While this section provides a rich array of direct participant quotations, it currently reads as an extended list of personal anecdotes rather than a thematically organised, reader-friendly presentation of findings. This approach makes it difficult to see how each quotation ties directly to the identified themes or subthemes, and it does not fully align with APA style guidelines for reporting qualitative results. Introduce all themes and subthemes at the start of the Results section, ideally with a visual representation showing their interrelationships.
We have re-organised the results section to clarify major themes and subthemes via headings. We have also included a table of themes (Table 2) at the start of the results section to guide the reader.

Begin each theme with a short analytic summary (1–3 sentences), followed by one or two representative quotes. Embed quotations within the narrative and provide interpretation immediately after each quote to link it back to the research questions or relevant literature.
We have re-structured the results section to ensure there is a short analytic summary at the start of each theme, and subtheme, as well as some interpretation of the quotes after their presentation. Further interpretation, including links to literature is presented within the discussion section, so we did not include this in the Results section to avoid duplication. In the results section, we also ensured that where necessary co-occurring neurodevelopmental conditions were acknowledged and discussed in relation to the subthemes.

Use concise, well-chosen excerpts rather than multiple long quotes on the same issue—this will reduce repetition and increase impact.
Literature applauds the use of longer quotes which effectively illustrate lived experience of the phenomenon under investigation ([Lingard 2019](https://doi.org/10.1007/s40037-019-00550-7)). However, we have reviewed all quotes and reduced (in length and quantity) where necessary. As the participant voice was central to the current research, we placed significant value in the raw complexities of participants lived experiences as presented within the results. Thus, deleting parts of quotes has the potential to undermine what mattered to participants.

Add demographic details after each quote (e.g., “Ella, female, 27, New Zealand”) to help readers interpret variation.
We have added the demographic details we have and can share to the names after each quote. As only one participant was based internationally, and was concerned about her family finding out about her interview. We therefore worked with her to understand what she would be comfortable with, and we agreed to not disclose her location. In addition, age was not collected.

Check for even distribution of quotes across participants; if some provided richer data, summarise rather than repeat multiple long extracts from the same individual.
We have checked for distribution across participants and removed some data from those who appeared more frequently. We have removed any repetition from the same individual in each subtheme. There are two participants (Danielle and Christine) who have much fewer quotes presented, however, the quality of interviews dictated to some extent the inclusion of quotes. However, we made sure that all participants were included in the final manuscript. Unfortunately, the two aforementioned interviews did not talk as freely as others, which limited their contribution to the development of themes.

Consider whether the word “treacherous” in the introductory sentence is the most appropriate descriptor.
We have changed treacherous to “*difficult*” for ease of clarity.

Discussion
Strengthen the link between discussion points and clearly defined research questions and themes/subthemes.
The discussion has been restructured to clearly map each subtheme to relevant literature. We have ensured all subthemes are present and discussed in relation to other studies.

You have mentioned the cross-cultural element but there is no reference to differences in the results or discussion – please elaborate for the reader
We apologise for the inclusion of this in the abstract. Unfortunately, there was only one international participant, so cross-cultural analysis and comparison was not possible. We have mentioned this in the discussion (limitations) section.

Where practical implications are discussed, provide more specific recommendations for interventions or policy.
We have made specific recommendations in relation to co-producing training, and re-organising pathways for assessment and support in DCD.

Include a brief reflexive statement on how researcher positionality may have influenced interpretation.
We have included a section on the research team and reflexivity at the end of the methods section.

Whilst you mention this in the limitations, some more information on how you unpacked the influence of the co-occurrences of DCD with ASD, ADHD would be helpful so that the reader can be confident that the findings are the result of DCD and not co-occurrences.
In the results section, we ensured that where necessary co-occurring neurodevelopmental conditions were acknowledged and discussed in relation to the subthemes. We have also checked the discussion section to ensure that all results discussed are relevant to DCD.

In the first paragraph, “explore” may be preferable to “reveal” to avoid implying preconceived bias.
We thank the reviewer for this suggestion. It has now been implemented.

Clarify the meaning of “transcending symptomology.”
We have changed the phrasing of this within text to “*extended beyond symptomology*”.

Limitations
Given that you used a diagnosis of DCD as your inclusion criteria, this should be discussed as a limitation as there is so little known about DCD and no nationally or internationally enforced guidelines for diagnosis. Whilst using the ADC is not perfect, it provides a standardised tool to ensure the ppts all meet the criteria for DCD in a research setting, discuss the implications of this criterion.
We agree the ADC could be useful in this context – we have included this within the limitations and future directions section.

**Reviewer 2**
Thank you for the opportunity to review this important manuscript. This manuscript makes an important contribution by documenting the lived experiences of adults with DCD. It highlights the significant role that lack of awareness and support from families, schools, workplaces, and broader society plays in shaping the quality of life, mood, and self-esteem of individuals with DCD. It also highlights important implications clinically but also for research and policy.
Strengths
- Provides valuable insight into the lived experience of adults with DCD, drawing on participants’ reflections across both childhood and adulthood.
- Brings needed attention to an under-researched area, with clear implications.

We thank the reviewer for their kind words.

That being said, the manuscript would benefit from revisions in some areas:
- Although the authors acknowledge that co-occurring neurodevelopmental (ND) conditions were present in approximately 50% of the sample, the analysis does not adequately disentangle the impact of DCD alone versus the cumulative impact of DCD plus other ND conditions.
- As a result, conclusions attributing outcomes such as cognitive load, concentration, and tiredness exclusively to DCD per se appear overstated. These domains may also be shaped independently or cumulatively by ADHD, autism, or mood symptoms. Tiredness/exhaustion can be attributed to mood symptoms as well which may be a consequence of experiencing DCD (and its impacts due to lack of awareness), or may be independent of DCD, or may be a result of the co-occurrence of DCD and other ND.
-The limitations section briefly notes this issue, but a more substantial engagement with the implications for interpreting results is warranted.
- Attribution of outcomes to DCD alone: Results and discussion sections sometimes present concentration, cognitive load, and tiredness as direct consequences of DCD. Given that 50% of participants reported co-occurring ND conditions (ADHD, autism, dyslexia), these effects may reflect cumulative neurodivergence. Please temper causal claims and/or clarify that these are participant attributions. Greater consideration of the potential role of co-occurring ND conditions in these areas would strengthen the validity of the conclusions.
- Handling of comorbidity: While noted in the limitations, comorbidity should be integrated more explicitly into interpretation of results. It may be beneficial to flag the quotes by comorbidity status after the name or providing sensitivity commentary to help readers judge which findings are more specific to DCD.
In the results section, we ensured that where necessary co-occurring neurodevelopmental conditions were acknowledged and discussed in relation to the subthemes. Co-occurrences were also acknowledged after every quote to ensure transparency. We have also checked the discussion section to ensure that all results discussed are relevant to DCD.

Major Issues
- Clarity of “lack of awareness”: In the abstract and early sections, it is initially unclear whether the “lack of awareness” refers to participants’ own awareness of DCD or the lack of awareness in their surrounding environments (families, schools, workplaces, and society). Clearer wording in the abstract and main body would prevent this ambiguity.
We thank the reviewer for highlighting this ambiguity. We have now amended lack of awareness to be “lack of societal awareness” for clarity.

- Claim of cultural differences: With only 10 participants from the UK and NZ, the study cannot claim meaningfully that cultural contexts were compared. This claim was not discussed in the discussion section either. Please adjust wording or clarify limitations.
Although we advertised the study to all English speaking countries, we were only able to recruit one individual from overseas (New Zealand). This participant grew up in the UK - we have not mentioned this as they wanted this to be redacted to ensure anonymity. They were therefore reflecting on experiences within the UK (childhood) and New Zealand (adulthood). One international participant was not enough to explore cross cultural differences, so we have removed all mention of this from the manuscript. We have added some blurb about this to the limitation section.

- Methods/Sample size rationale: Ten interviews are appropriate. However, a brief rationale may be required (e.g., information power, iterative analysis) as well as a statement on how saturation was judged to justify why 10 participants were included (and not more or less).
We have now included this information in the methods section alluding to previous literature that suggests 10 interviews for lived experience is sufficient. We have also mentioned data saturation, and reflexivity.

Minor Issues
- Several typos, grammatical errors, and issues with punctuation were noted. A careful proof-read is recommended.
We have now proof-read the manuscript to ensure typos, grammatical and punctuation errors are resolved.

- Abbreviations were noted that were not written in full in the first instance (Eg EACD) and would be helpful for the reader if presented appropriately in the manuscript.
We thank the reviewer for highlighting this omission, we have now made sure all acronyms are written out in full before they appear in text.

- Use of causal language should be reconsidered as DCD may not be the only reason for the cognitive load/tiredness.
We have amended the language in both the results and discussion to mitigate for implied causality. Rather the changes allude to relationships shown in the literature.
