# Supplementary material for: ‘The world is just so fast, and I’m not fast… it’s just really, really difficult to keep up’: A qualitative exploration of the lived experience of adults with Developmental Coordination Disorder
Source: PLoS One. 2026 May 28;21(5):e0350273. doi: 10.1371/journal.pone.0350273 (PMC13218497; doi:10.1371/journal.pone.0350273)
Supplement: Attachment — Submitted filename: Review Comments.docx [file pone.0350273.s003.docx]

Review Comments

Thank you for your openness and for addressing the comments. While several comments have been addressed and efforts have been made to adequately address these, some things still require addressing.

1. Abstract

A. Methods - Minor Comment

“Participants were recruited both nationally and internationally Lived experience interviews focused on experiences in primary care, education, friendships, wellbeing, employment and romantic relationships.”

Needs a period/full stop between “internationally” and “lived”

B. Methods – Major Comment

Please provide some demographic information about participants – males, females, mean age, etc.

I understand from the response to the other reviewer that some demographic data were not collected and thus mean age and SD cannot be provided. While the absence of age data is acknowledged in the Limitations section, it will also benefit this manuscript to acknowledge this early on in the methods section. This is particularly important given the manuscript’s emphasis on lifespan and developmental transitions. As such, it would be important to signal this constraint earlier (e.g., in Methods or when interpreting age-related experiences), or to further temper claims that rely on age or generational differences throughout the manuscript.

For example – it can be briefly acknowledged in Methods or Participants that participant age was not systematically collected, and therefore age-related analyses or generational interpretations are limited.

C. Results – major comment

“Emergent themes highlighted a major lack of societal awareness in all life domains, and difficulty navigating health, education and workplace systems for support, resulting in relative abandonment and a lack of validation within their lived experience of DCD.”

Thank you for addressing the earlier comment around this statement. This statement would benefit from more clarity. Presently, it is not clear from this statement who is impacted or experiences the difficulty navigating these systems. Is it difficulty for those with DCD or society. My understanding was low societal awareness, but difficulties were experienced by individuals with DCD.

2. Introduction

A. Minor comments – several areas need proof-reading and editing for grammatical accuracy. Eg: a) “it is also known ~~as~~ and referred to [as] Dyspraxia”. b) After references 6-8 there should be a space between the period and the next sentence starting with “The”. c) “Despite between 30-70% of children [being] diagnosed with DCD”. d) “with studies highlighting that these challenges extended beyond the ~~t~~ motor skills”. e) with tasks such as cooking and cleaning, leaving adults”

B. Major comments – “It is, however, important to note that DCD is often diagnosed with a co-occurring condition, such as Autism (50%) and ADHD (30%) [16], more research is needed to disentangle the role DCD plays in these cognitive challenges.”

Thank you for attempting to clarify that executive functioning challenges in DCD could be impacted by DCD but also by co-occurring NDC. Unfortunately, this statement is not very clear and will benefit from restructuring. Secondly, I’m wondering if this is an appropriate place for this statement. if the manuscript is not intending to resolve this question of disentangling the role of DCD in these cognitive challenges, it should likely not be stated in this way in the introduction. It would be enough to acknowledge that DCD is well-known to co-occur with other neurodevelopmental conditions, eg, ASD, ADHD. Thirdly, and importantly, sometimes there are multiple co-occurring conditions alongside DCD, not just one other co-occurring condition. So, the language here should be clearer.

**D. Additional major comment – analytic rationale**
Please consider strengthening the rationale for the study in the latter part of the introduction. While the importance of DCD across the lifespan is discussed, the specific analytic contribution of the current qualitative study (beyond documenting lived experience) could be articulated more clearly. For example, clarifying what gaps in the existing qualitative literature this study addresses, and how it advances understanding beyond previous work, would strengthen the framing of the paper.

3. Methods

A. “The sample comprised 8 females and 2 males and 5 had a co-occurrence (two with Autism, two with Dyslexia, and one with ADHD).”

It would be good to include where/how these co-occurring diagnoses made, or who made them and at what age if this information is available. Was any evidence of these diagnoses assessed or gathered by the researchers? If not, then this should be acknowledged in the methods section. Such as, self-reported diagnoses, not verified independently by researchers, etc.

B. Procedure – “Interviews lasted between 30 minutes to an hour.” In the abstract it states 30-45 minutes. Please consider consistency with the information throughout the manuscript.

C. Ages – As noted above, I understand from the response to the other reviewer that demographic data were not collected and thus mean age and SD cannot be provided. Please acknowledge this constraint explicitly in this section. Doing so only in the limitations section may not be adequate. For example – it can be briefly acknowledged in Methods or Participants that participant age was not systematically collected, and therefore age-related analyses or generational interpretations are limited.

4. Results

A. “The current analysis presents the difficult road travelled by adults with DCD both contemporaneously and retrospectively. The data transcends symptomology to wider system issues, including lack of societal awareness and effective support which impact the lived experience of health and wellbeing within the DCD diagnosis. Stories of abandonment, struggles to be heard, and battles to access effective support across sectors, collectively revealed a society which is failing to scaffold vulnerable adults, and by extension, children”

This paragraph would be more appropriate in the discussion section as it draws all of the themes together and is more conclusive. The results section should ideally be only stating the results of the study, i.e., the themes and sub-themes that came about from the raw data as has been done wonderfully in the remainder of the section.

B. Theme 1 - “in rites of passage”. It may be more appropriate to state this as lifecycle transitions or stereotypical/cultural developmental stages.

C. Subtheme 1.2: “Belittling”

C.1. It may not be appropriate to use emotive terms e.g. “belittling” unless that was what was specifically reported by participants. Eg; One participant reported “I felt belittled”. It is preferable to use neutral language when summarising both in the heading and in-text where the word belittling has been applied. Example of appropriate use – “some participants drew upon the label ‘clumsy’ on reflecting upon their experiences in childhood”.

C.2. “However, the reality of their ‘clumsiness’ sometimes resulted in direct implications for their health and wellbeing, as well as scrutiny for their family.”

It may be helpful to more clearly distinguish between participants’ self-descriptions and the authors’ analytic framing. Additionally, statements referring to “direct implications for health and wellbeing” and “scrutiny for their family” are somewhat broad and would benefit from clarification or more precise description of the nature and source of these impacts. Adopting more neutral, descriptive language and specifying the mechanisms or contexts involved may strengthen clarity and interpretability.

D. Subtheme – “rites of passage”

Please consider using more appropriate terminology such as functional milestones, developmental stages, lifecycle transitions, etc than rites of passage.

The discussion of driving-related difficulties would benefit from more cautious interpretation. While the authors appropriately note the presence of co-occurring ADHD in one participant, the subsequent attribution of driving challenges to cognitive demands (e.g., attention, navigation, spatial awareness) may extend beyond what can be supported by the qualitative data presented. It may be helpful to clarify that these interpretations reflect participants’ reported experiences rather than inferred mechanisms, and to avoid implying that cognitive difficulties are independent of, or separable from, motor coordination challenges. Additionally, greater caution may be warranted when generalising across participants with and without co-occurring conditions.

E. Subtheme Cognitive Load

“The cognitive load associated with driving was also seen as problematic in relation to planning and processing more broadly. Participants revealed the reality of life within which taken for granted practices demanded significant efforts.”

“Additionally, the cognitive challenges participants with DCD experienced, sometimes left them feeling exhausted, even after ‘simple’ tasks.”

Please consider using more cautious interpretation and language than conclusive/causal language. Eg: participant-reported cognitive challenges.

F. Subtheme Commonality in difference in adult friendships

The language in this section is relatively abstract and interpretive. Greater specificity regarding participants’ reported experiences of peer relationships, and clearer explanation of terms such as “commonality in difference,” would improve clarity and readability.

G. Subtheme 1.7: Commonality in difference in romantic relationships

Please consider correcting the formatting between the paragraph and the heading. Please also consider explaining “commonality in difference” in this section as well to clarify meaning.

H. Subtheme 1.8: Lack of equity in employment opportunities

The interpretive framing may benefit from greater caution and clarity. In particular, while participants often attributed workplace challenges to DCD, a substantial proportion of the quoted examples involve individuals with co-occurring neurodevelopmental conditions (e.g., ADHD, Autism, Dyslexia). Given this, it may be helpful to more explicitly acknowledge the potential influence of co-occurring conditions and to avoid attributing these experiences primarily to DCD alone. Clarifying that these interpretations reflect participants’ perceptions, rather than definitive causal attribution, would strengthen the analytic transparency. Additionally, the reference to “lack of equity in employment opportunities” could be further specified or supported, as it currently reads as a broad conclusion relative to the qualitative evidence presented.

I. “Participants often felt that there was a lack of interest and investment in DCD at a societal level, which impacted them on an individual level, leaving some frustrated and tired from continually advocating for themselves.”

This would be stronger if framed in terms of participant-reports rather than “participants felt” for a less emotive, more neutral framing.

J. Subtheme 2.8: Inherent lack of awareness compared to other neurodevelopmental conditions

Both paragraphs would benefit from greater clarity around who experiences the lack of awareness is, and where the lack of awareness is observed. As it currently stands, the statements are vague.

Eg: “All participants expressed a profound lack of awareness of DCD, especially when compared to other NDCs.”

K. Subtheme 2.9: Huge hole in community level support and advocacy

Please consider reframing “huge hole” which comes across as colloquial. Consider “gap” or “considerable gap” instead.

“participants felt a sense of abandonment” please consider instead “participants reported a sense…”

L. Participant voice vs interpretation

Across several subthemes, there are instances where analytic language appears to move beyond participant descriptions into broader interpretation (e.g., societal failure, abandonment, inequity). Please consider more consistently signalling when statements reflect participant-reported experiences versus author interpretation. Explicitly distinguishing between these may strengthen analytic clarity.

M. Co-occurring conditions across themes

Please consider more consistently acknowledging the presence of co-occurring neurodevelopmental conditions across subthemes, rather than addressing this only in selected sections. Given the prominence of co-occurrence within the sample, a brief reminder within relevant subthemes may help avoid over-attribution of experiences solely to DCD.

5. Discussion

A. Please consider reframing emotive language to more neutral language, eg: “belittling”.

B. Please consider reframing “alluded” throughout the transcript to “reported”, “recounted”, “identified” etc.

C. Please consider reframing “rites of passage” to “functional milestones”, “developmental stages”, etc.

D. Please consider throughout the discussion section including existing qualitative research where similar themes have or have not been considered or addressed previously, and whether these themes are upheld or challenged relative to previous research, even if lifespan or childhood research. If none is available – this can also be acknowledged in the discussion section.

E. “appeared to suffer at the hands”. Please consider using neutral language rather than emotive language.

F. Causal and diagnostic implications

Please consider reviewing the discussion section holistically to ensure that language does not imply causal relationships or diagnostic utility that cannot be supported by the qualitative design. In several places, emotional experiences and functional challenges are discussed in ways that suggest DCD as a primary explanatory mechanism. Greater caution in attributing causality, and clearer framing of findings as participant-reported associations and interpretations, would strengthen the discussion.

G. Diagnostic relevance

Please consider tempering statements that suggest findings may directly support identification or diagnostic processes for DCD in adulthood. While the experiences described are clearly important, stronger empirical or theoretical justification would be required to support claims relating to diagnostic utility.

6. Limitations

A. co-morbid Autism – please consider using the term co-occurring.

B. “Although this reflects the reality of DCD diagnoses [16] in places it was challenging to disentangle causation, resulting in an element of speculation.”

Please reconsider this statement, a qualitative study cannot comment on causation regardless. This sentence would benefit from greater clarity and precision. The phrasing is somewhat vague and may understate an important methodological limitation. It may be helpful to more explicitly acknowledge the limits of causal interpretation inherent in the study design and to clarify where and how interpretive uncertainty arose. Please also state how future research could address this and in what way.

While this issue is acknowledged in the limitations, similar causal or attributional language appears elsewhere in the manuscript, and addressing this solely within the limitations section may not be sufficient.

C. “and we were interviewing adults who may not have been able to access such historic evidence.” This is speculative as the researchers do not know whether or not the participants had access to this information.

D. Scope of inference

Please consider explicitly stating that the findings reflect the experiences of a small qualitative sample and may not be representative of all adults with DCD. Clarifying the scope and transferability of the findings would further strengthen this section.

7. Conclusion

A. “Individuals with DCD often feel alone, isolated and misunderstood” Please consider reframing to “often report feeling…”

B. “Data revealed that there is an urgent need for the profile of DCD to be raised” Before this statement please summarise the actual results of the data, i.e., data revealed themes and subthemes around different aspects of life, for example, … followed by the implication that these data indicate a need to raise the profile.

Unfortunately, the conclusion section requires more specificity as it is currently somewhat vague about the results and conclusions.

C. Alignment with data

Please consider ensuring that all concluding statements are clearly traceable to the themes and subthemes presented in the Results section. Where broader implications are drawn, it may be helpful to explicitly signal these as interpretations or implications rather than direct findings.

Overarching comment

While specific comments are provided throughout, my overarching comments and concerns relate to analytic framing across the manuscript, particularly the distinction between participant-reported experiences and author interpretation, the handling of co-occurring neurodevelopmental conditions, and the avoidance of causal or diagnostic implications. Addressing these issues holistically would substantially strengthen the manuscript.
