# Supplementary material for: ‘The world is just so fast, and I’m not fast… it’s just really, really difficult to keep up’: A qualitative exploration of the lived experience of adults with Developmental Coordination Disorder
Source: PLoS One. 2026 May 28;21(5):e0350273. doi: 10.1371/journal.pone.0350273 (PMC13218497; doi:10.1371/journal.pone.0350273)
Supplement: Attachment — Submitted filename: Response_to_reviewers_auresp_2.docx [file pone.0350273.s005.docx]

**Response to reviewers**

**Reviewer 1**

This is a thoughtful, and much-needed contribution to the literature on adult DCD. With modest refinements the paper will be well positioned to inform research, practice, and policy, while remaining true to the lived experiences at its core. The findings are shared with care, reflexivity, and ethical sensitivity. Participant quotations are clear and emotionally resonant, effectively conveying experiences of frustration, invalidation, and systemic neglect. The thematic structure is now clearer and more reader-friendly and the discussion of societal awareness and systemic abandonment is a strong addition.
We are sincerely grateful to the reviewer for their generous and thoughtful feedback. It is encouraging to know that the revisions made in the previous round have strengthened the manuscript in these important areas, and we have endeavoured to build upon this foundation in the current revision.

However, there are still some areas that need attention before ready for publication.
At present, the manuscript sometimes implies that DCD is uniquely neglected compared to other neurodevelopmental conditions. Whilst I agree with this, it would be good to unpack which systems (healthcare, education, employment, community) are most inequitable, and whether this reflects absolute neglect or relative invisibility compared to conditions such as ADHD or ASD.

We appreciate the reviewer raising this nuanced and important point, and we agree that unpacking systemic inequity in greater depth would be a valuable contribution. However, we respectfully note that the qualitative design and data collected in the current study do not allow for systematic comparison across sectors in terms of the relative degree of inequity experienced. Whilst the results section does address the lack of awareness and support across healthcare, education, and the wider community, we feel that drawing comparisons between sectors would risk extending beyond what the data can meaningfully support. In the discussion, we have referenced existing literature that speaks to differential levels of awareness and support across neurodevelopmental conditions, and we feel this provides appropriate contextualisation without overstating the interpretive reach of the current findings.

Additionally, as approximately half of the participants reported co-occurring neurodevelopmental conditions, the analysis and interpretation at times appears to attribute outcomes such as cognitive load, concentration difficulties, fatigue, and executive functioning challenges primarily to DCD. Greater interpretive caution is required in these areas and it would be helpful to more explicitly distinguish between participant-reported attributions and condition-specific effects, and to integrate consideration of co-occurring conditions more consistently throughout the Results and Discussion sections rather than primarily within the limitations. This would strengthen the validity and transparency of the conclusions.

We agree that greater and more consistent interpretive caution was warranted throughout the manuscript. In response, we have made a concerted effort to revise the language across both the Results and Discussion sections to ensure that all data is clearly framed as reflecting participant-reported experiences rather than researcher-inferred causal mechanisms. We feel it is important to present participants' own attributions of their challenges to DCD, particularly given that cognition was not included as an interview prompt and yet emerged organically and consistently across all participant accounts - a finding which we feel is itself noteworthy and worthy of discussion. Nonetheless, we fully appreciate the need for analytic transparency regarding co-occurring conditions, and have therefore included explicit acknowledgement of the potential role of co-occurring diagnoses within each relevant subtheme in the Results section, and have woven this consideration throughout the Discussion to ensure the reader is consistently equipped to interpret the findings with appropriate caution.

The paper is framed as taking a lifespan and cross-cultural approach. However, the empirical basis for cross-cultural analysis is not justified, as the sample is predominantly UK-based, with only one participant residing overseas, the study cannot reasonably be framed as international, and this wording should be amended throughout. Similarly, while participants retrospectively reflect on childhood and adulthood experiences, the paper would benefit from greater clarity regarding how a “lifespan approach” is operationalised analytically. Explicitly articulating how experiences across different developmental stages were examined would improve conceptual clarity.
Upon reflection, we agree that framing the study as adopting a lifespan or cross-cultural approach was not adequately supported by the empirical basis of the sample. As a result, we have removed all references to a lifespan approach throughout the manuscript. The sole reference to cross-cultural considerations that remains appears in the Limitations section, where we acknowledge that cross-cultural comparison was not possible given that only one participant was based internationally - a limitation that was flagged by previous reviewers and which we felt was important to retain for transparency.

Abstract
Even though you do not have the ages of the ppts, I would tell the reader their sex in the abstract
We thank the reviewer for this suggestion and agree that including participant sex in the abstract provides useful contextual information for the reader. We have now added this information to the abstract accordingly.

Intro
The opening section needs to more clearly establish that DCD is fundamentally a movement condition; this is not sufficiently explicit in the initial description. Although the four DSM-5 criteria are listed, this approach is overly basic and reads at an undergraduate level rather than reflecting the conceptual depth expected in a published journal article. A more sophisticated, narrative description of the condition is needed.

This section was modified to include the DSM-5 criteria in response to the previous round of revisions, however we have now revised it further to incorporate both a nuanced narrative description of DCD as a fundamentally movement-based condition and a more conceptually integrated presentation of the DSM-5 criteria.

The introduction should be written in tight APA style, with only essential information included. Currently, key messages are diluted by unnecessary dialogue. For example, the sentence “This is also reflected in academic literature” is redundant and should be removed, with the relevant references cited directly.
While the manuscript states that DCD manifests in adulthood, greater detail is required to explain how this occurs. The paragraph would benefit from clearer elaboration of adult impacts, such as ongoing difficulties in education (e.g. handwriting and fine motor skills in higher education) and challenges with independent living skills.

We have carefully reviewed the introduction and removed unnecessary dialogue to ensure the writing is concise and appropriately focused. We have also expanded the discussion of how DCD manifests in adulthood, including greater elaboration on the impact of ongoing motor difficulties in higher education and on independent living skills, ensuring that the adult experience of DCD is more clearly and substantively conveyed from the outset of the paper.

References are frequently incorrectly positioned throughout the introduction:
• Citations should follow the evidence presented, not appear at the beginning of sentences.
• Several references are missing spaces between the citation and subsequent text.
• Phrases such as “Previous literature” should be followed by citations, not preceded by them. For example, sentences beginning with bracketed references (e.g. “[14] Previous literature…”, “[18] This study therefore aimed…”) should be revised for correctness and readability.

There are repeated issues with the method of citation, where prior research is not properly embedded within the narrative. For example:
“[6,20][14] Previous research has alluded to individuals with DCD finding learning to drive challenging [14].”This structure is awkward and does not meaningfully integrate the literature into the discussion.

The paragraph on inequitable support across neurodevelopmental conditions is conceptually strong but poorly referenced. The standalone list of citations ([51–53][64][70][51–53]) is unclear and must be explicitly linked to the preceding claims about awareness, validation, and lived experience.

We apologise for these formatting inconsistencies, which arose from technical issues with the reference management software used during manuscript preparation. We have now thoroughly reviewed and corrected all in-text citations throughout the manuscript to ensure they are consistently and accurately positioned.

The discussion of prior qualitative research needs refinement. While the manuscript states that existing qualitative studies focus on narrow aspects of adulthood (e.g. university life or driving), the authors should check whether additional qualitative studies exist and ensure the claim is accurate and well-supported.

We thank the reviewer for prompting us to revisit this claim. In response, we conducted an updated literature review and identified one additional qualitative study published after the original submission of this manuscript. This paper has now been incorporated into the introduction, and we have taken care to more clearly articulate the specific gaps in the existing qualitative literature that the current study addresses.

Methods
The statement that participants were recruited by contacting “all English-speaking national DCD charities globally” requires quantification and clarification. The authors should specify how many charities were contacted, In which countries, and avoid overstating the global reach of recruitment, as the sample does not reflect this claim.
We have amended the manuscript to specify that four national DCD charities were contacted, along with the continents in which they are based. We have refrained from specifying individual countries, as doing so could risk compromising participant anonymity given the limited DCD infrastructure in some regions.

The inclusion of a table listing interview questions may read more smoothly if these are integrated into prose, rather than presented in tabular form.

We were asked by previous reviewers to include this information in tabular form. As writing these in prose will not fundamentally change the content, we have decided to keep the table in the manuscript.

Procedure
When stating that “Participants were asked seven questions, with prompts for further discussion used where necessary”, please remind the reader where to locate these questions in the paper.

We have now added (see Table 1) to the manuscript for ease.

Results
Although the table of themes is informative, it currently attempts to do too much. Standard qualitative reporting practice would involve presenting a thematic map or list of themes only, and introducing example data later within the detailed analysis. Revising the table to function purely as a thematic overview would improve clarity and help readers better contextualise the findings.

We have now removed the example data from this table for ease of interpretation.

Discussion
Claims that the findings apply across the lifespan are overstated. While the study includes reflections on childhood and adulthood, it only briefly addresses selected aspects of the lifespan and should be framed more cautiously.

We agree and, as noted above, have removed all references to a lifespan approach throughout the manuscript.

In several places, references are cited without sufficient explanation or integration into the discussion. For example, the statement “similarly to previous research, motor skill challenges in childhood (subtheme 1.1) were seen to play a central role in the experiences of individuals with DCD [8]” is too abstract; the cited work needs to be more clearly explained and explicitly linked to the current findings.

Similarly, when discussing cognitive load and driving, the sentence referencing anxiety (“…which was anxiety inducing [36]”) lacks context. The reader needs to understand how reference [36] relates conceptually or empirically to the present findings.

We have been through the manuscript to ensure all cited literature is explained in relation to the current study.

In the section on physical activity, childhood social exclusion is well described; however, the discussion should also briefly acknowledge the health implications of reduced physical activity in adulthood, including increased risks related to obesity and cardiovascular health, which are concerns for adults with DCD.

We are grateful to the reviewer for raising this important point, which adds meaningful depth to the discussion of physical activity in the context of DCD. We have now incorporated a sentence acknowledging the health implications of reduced physical activity in adulthood, including the increased risk of obesity and cardiovascular disease, accompanied by appropriate supporting references.

Finally, ensure terminological accuracy and consistency throughout; the correct diagnostic label is DSM-5, not DSM-V.
We have now updated this in the manuscript and ensure all acronyms are accurate throughout.

Minor Points
• Some sections would benefit from tighter language to reduce repetition.
• Ensure consistent use of terminology when discussing inequity versus invisibility.
• A final proofread for flow and concision in the discussion would further strengthen readability.
We have carefully proofread the manuscript in its entirety, addressing areas of repetition, improving overall flow, and ensuring terminological consistency throughout.

• Some constructs (e.g., “cognitive load,” “executive functioning”) would benefit from clearer definition. Clarifying whether these terms are used descriptively (as participant experiences) or in reference to established cognitive constructs would improve precision.

We have revised the relevant sections of both the Results and Discussion to clearly signal when these terms are being used descriptively to reflect participant-reported experiences, and when they are being used in reference to established theoretical or cognitive constructs.

• Age data were not collected, which limits interpretation of generational effects. While this is acknowledged as a limitation, its implications for interpreting retrospective accounts could be more explicitly discussed.

We agree with the reviewer that the absence of age data warranted more substantive discussion than was provided in the original manuscript. We have therefore expanded the relevant section in the Limitations to more explicitly address how the lack of age data constrains our ability to situate participants' retrospective accounts within specific historical or generational contexts, to identify whether variations in experience reflect generational differences or individual variation, and to determine how the passage of time between participants' experiences and the interview may have affected both memory accuracy and narrative framing. We have also noted that future research would benefit from the collection of age data to enable more nuanced cohort-level interpretation.

**Reviewer 2**

Thank you for your openness and for engaging with the previous review. While several comments have been addressed and clear efforts have been made to respond, a number of substantive issues remain that require further consideration.
“Participants were recruited both nationally and internationally Lived experience interviews focused on experiences in primary care, education, friendships, wellbeing, employment and romantic relationships.” Needs a period/full stop between “internationally” and “lived”

Thank you for highlighting this omission. We have included this and proofread the paper.

B. Methods – Major Comment
Please provide some demographic information about participants – males, females, mean age, etc. I understand from the response to the other reviewer that some demographic data were not collected and thus mean age and SD cannot be provided. While the absence of age data is acknowledged in the Limitations section, it will also benefit this manuscript to acknowledge this early on in the methods section. This is particularly important given the manuscript’s emphasis on lifespan and developmental transitions. As such, it would be important to signal this constraint earlier (e.g., in Methods or when interpreting age-related experiences), or to further temper claims that rely on age or generational differences throughout the manuscript. For example – it can be briefly acknowledged in Methods or Participants that participant age was not systematically collected, and therefore age-related analyses or generational interpretations are limited.

We agree with the reviewer that early acknowledgement of the absence of age data is important, particularly given the developmental focus of the research. We have now included an explicit statement within the Participants section of the Methods acknowledging that participant age was not systematically collected and that, as a consequence, age-related analyses and generational interpretations are limited. We believe that flagging this constraint early in the manuscript, rather than solely in the Limitations, better prepares the reader to interpret the findings with appropriate caution.

C. Results – major comment
“Emergent themes highlighted a major lack of societal awareness in all life domains, and difficulty navigating health, education and workplace systems for support, resulting in relative abandonment and a lack of validation within their lived experience of DCD.” Thank you for addressing the earlier comment around this statement. This statement would benefit from more clarity. Presently, it is not clear from this statement who is impacted or experiences the difficulty navigating these systems. Is it difficulty for those with DCD or society. My understanding was low societal awareness, but difficulties were experienced by individuals with DCD.

We are grateful to the reviewer for highlighting this ambiguity. We agree that the original wording did not clearly convey that it was individuals with DCD, rather than society more broadly, who experienced difficulties navigating these systems. We have amended the sentence to read: *"Emergent themes highlighted a major lack of societal awareness in all life domains, which often led to participants facing difficulties navigating health, education and workplace systems for support, resulting in relative abandonment and a lack of validation within their lived experience of DCD."*

2. Introduction
A. Minor comments – several areas need proof-reading and editing for grammatical accuracy. Eg: a) “it is also known as and referred to [as] Dyspraxia”. b) After references 6-8 there should be a space between the period and the next sentence starting with “The”. c) “Despite between 30-70% of children [being] diagnosed with DCD”. d) “with studies highlighting that these challenges extended beyond the t motor skills”. e) with tasks such as cooking and cleaning, leaving adults”
All of the instances highlighted have been corrected, and we have conducted a comprehensive proofread of the full manuscript to ensure no similar errors remain.

B. Major comments – “It is, however, important to note that DCD is often diagnosed with a co-occurring condition, such as Autism (50%) and ADHD (30%) [16], more research is needed to disentangle the role DCD plays in these cognitive challenges.” Thank you for attempting to clarify that executive functioning challenges in DCD could be impacted by DCD but also by co-occurring NDC. Unfortunately, this statement is not very clear and will benefit from restructuring. Secondly, I’m wondering if this is an appropriate place for this statement. if the manuscript is not intending to resolve this question of disentangling the role of DCD in these cognitive challenges, it should likely not be stated in this way in the introduction. It would be enough to acknowledge that DCD is well-known to co-occur with other neurodevelopmental conditions, eg, ASD, ADHD. Thirdly, and importantly, sometimes there are multiple co-occurring conditions alongside DCD, not just one other co-occurring condition. So, the language here should be clearer.

This was added in at the request of a previous reviewer, however, we agree that the statement as originally worded was both unclear and potentially misleading in terms of the scope of the current manuscript. As the study was not designed to disentangle the relative contributions of DCD and co-occurring conditions to cognitive challenges, we have removed the reference to a need for further research in this area from the introduction. We have instead retained an acknowledgement that DCD frequently co-occurs with other neurodevelopmental conditions, including Autism and ADHD, and have taken care to reflect the reality that multiple co-occurring diagnoses are possible.

D. Additional major comment – analytic rationale
Please consider strengthening the rationale for the study in the latter part of the introduction. While the importance of DCD across the lifespan is discussed, the specific analytic contribution of the current qualitative study (beyond documenting lived experience) could be articulated more clearly. For example, clarifying what gaps in the existing qualitative literature this study addresses, and how it advances understanding beyond previous work, would strengthen the framing of the paper.
We have revised the concluding section of the introduction to more clearly identify the gaps that the study addresses, including the limited qualitative attention given to romantic relationships, adult friendships, and retrospective adult accounts of experiences in healthcare and education.

3. Methods
A. “The sample comprised 8 females and 2 males and 5 had a co-occurrence (two with Autism, two with Dyslexia, and one with ADHD).” It would be good to include where/how these co-occurring diagnoses made, or who made them and at what age if this information is available. Was any evidence of these diagnoses assessed or gathered by the researchers? If not, then this should be acknowledged in the methods section. Such as, self-reported diagnoses, not verified independently by researchers, etc.

We have added an explicit statement to the Methods section clarifying that all diagnoses, including both DCD and any co-occurring conditions, were self-reported by participants and were not independently verified by the research team.

B. Procedure – “Interviews lasted between 30 minutes to an hour.” In the abstract it states 30-45 minutes. Please consider consistency with the information throughout the manuscript.

Having reviewed the interview data, we can confirm that interviews lasted between 30 minutes and one hour, and have amended the abstract accordingly to ensure consistency across the manuscript.

C. Ages – As noted above, I understand from the response to the other reviewer that demographic data were not collected and thus mean age and SD cannot be provided. Please acknowledge this constraint explicitly in this section. Doing so only in the limitations section may not be adequate. For example – it can be briefly acknowledged in Methods or Participants that participant age was not systematically collected, and therefore age-related analyses or generational interpretations are limited.

We have now included this in the participants section of the methods.

4. Results
A. “The current analysis presents the difficult road travelled by adults with DCD both contemporaneously and retrospectively. The data transcends symptomology to wider system issues, including lack of societal awareness and effective support which impact the lived experience of health and wellbeing within the DCD diagnosis. Stories of abandonment, struggles to be heard, and battles to access effective support across sectors, collectively revealed a society which is failing to scaffold vulnerable adults, and by extension, children”. This paragraph would be more appropriate in the discussion section as it draws all of the themes together and is more conclusive. The results section should ideally be only stating the results of the study, i.e., the themes and sub-themes that came about from the raw data as has been done wonderfully in the remainder of the section.
We thank the reviewer for this constructive observation and agree that this passage was more interpretive and conclusive in nature than is appropriate for a Results section. We have amended the opening of the Results section so that it now simply and clearly introduces the number of major themes and subthemes identified, in keeping with standard qualitative reporting practice. The interpretive framing previously included in this passage has been moved to the opening of the Discussion section, where it more appropriately contextualises the findings for the reader.

B. Theme 1 - “in rites of passage”. It may be more appropriate to state this as lifecycle transitions or stereotypical/cultural developmental stages.
D. Subtheme – “rites of passage”
Please consider using more appropriate terminology such as functional milestones, developmental stages, lifecycle transitions, etc than rites of passage.

We have amended this terminology throughout the manuscript to "developmental milestones which enable independence," which we feel more accurately and neutrally captures the nature of the experiences described by participants.

C. Subtheme 1.2: “Belittling”
C.1. It may not be appropriate to use emotive terms e.g. “belittling” unless that was what was specifically reported by participants. Eg; One participant reported “I felt belittled”. It is preferable to use neutral language when summarising both in the heading and in-text where the word belittling has been applied. Example of appropriate use – “some participants drew upon the label ‘clumsy’ on reflecting upon their experiences in childhood”.
We agree that the term "belittling" was not directly drawn from participant language and was therefore not appropriately grounded in the data. We have revised the subtheme title and all associated in-text references to use more neutral, descriptive language, renaming the subtheme "Acquiring the label of clumsy".

C.2. “However, the reality of their ‘clumsiness’ sometimes resulted in direct implications for their health and wellbeing, as well as scrutiny for their family.”
It may be helpful to more clearly distinguish between participants’ self-descriptions and the authors’ analytic framing. Additionally, statements referring to “direct implications for health and wellbeing” and “scrutiny for their family” are somewhat broad and would benefit from clarification or more precise description of the nature and source of these impacts. Adopting more neutral, descriptive language and specifying the mechanisms or contexts involved may strengthen clarity and interpretability.

We have revised the relevant passage to more clearly distinguish between participant-reported experiences and researcher interpretation and have replaced broad or imprecise language with more specific, descriptive framing that more closely reflects what participants described. We have also ensured that the language is non-causational throughout this section.

The discussion of driving-related difficulties would benefit from more cautious interpretation. While the authors appropriately note the presence of co-occurring ADHD in one participant, the subsequent attribution of driving challenges to cognitive demands (e.g., attention, navigation, spatial awareness) may extend beyond what can be supported by the qualitative data presented. It may be helpful to clarify that these interpretations reflect participants’ reported experiences rather than inferred mechanisms, and to avoid implying that cognitive difficulties are independent of, or separable from, motor coordination challenges. Additionally, greater caution may be warranted when generalising across participants with and without co-occurring conditions.
E. Subtheme Cognitive Load
“The cognitive load associated with driving was also seen as problematic in relation to planning and processing more broadly. Participants revealed the reality of life within which taken for granted practices demanded significant efforts.”
“Additionally, the cognitive challenges participants with DCD experienced, sometimes left them feeling exhausted, even after ‘simple’ tasks.”
Please consider using more cautious interpretation and language than conclusive/causal language. Eg: participant-reported cognitive challenges.

F. Subtheme Commonality in difference in adult friendships
The language in this section is relatively abstract and interpretive. Greater specificity regarding participants’ reported experiences of peer relationships.
H. Subtheme 1.8: Lack of equity in employment opportunities
The interpretive framing may benefit from greater caution and clarity. In particular, while participants often attributed workplace challenges to DCD, a substantial proportion of the quoted examples involve individuals with co-occurring neurodevelopmental conditions (e.g., ADHD, Autism, Dyslexia). Given this, it may be helpful to more explicitly acknowledge the potential influence of co-occurring conditions and to avoid attributing these experiences primarily to DCD alone. Clarifying that these interpretations reflect participants’ perceptions, rather than definitive causal attribution, would strengthen the analytic transparency. Additionally, the reference to “lack of equity in employment opportunities” could be further specified or supported, as it currently reads as a broad conclusion relative to the qualitative evidence presented.
L. Participant voice vs interpretation
Across several subthemes, there are instances where analytic language appears to move beyond participant descriptions into broader interpretation (e.g., societal failure, abandonment, inequity). Please consider more consistently signalling when statements reflect participant-reported experiences versus author interpretation. Explicitly distinguishing between these may strengthen analytic clarity.
As per the above we have changed all language to be participant-centred and reflective of their experiences. We have also included discussion regarding the co-occurrence of other NDCs throughout the discussion where appropriate to ensure the reader can make an informed interpretation of the data. We have moved all author interpretation to the discussion.

G. Subtheme 1.7: Commonality in difference in romantic relationships
Please consider correcting the formatting between the paragraph and the heading.
We have amended the formatting, and proofread the manuscript

Please also consider explaining “commonality in difference” in this section as well to clarify meaning.
We have added a brief clarifying sentence to explain the concept of "commonality in difference" within this section, specifying that this refers to participants' reported capacity to form meaningful connections with others who shared experience of neurodivergence or difference.


I. “Participants often felt that there was a lack of interest and investment in DCD at a societal level, which impacted them on an individual level, leaving some frustrated and tired from continually advocating for themselves.”
This would be stronger if framed in terms of participant-reports rather than “participants felt” for a less emotive, more neutral framing.
We have now amended the language as suggested by the reviewer, and thank them for strengthening the framing of this sentence.

J. Subtheme 2.8: Inherent lack of awareness compared to other neurodevelopmental conditions
Both paragraphs would benefit from greater clarity around who experiences the lack of awareness is, and where the lack of awareness is observed. As it currently stands, the statements are vague. Eg: “All participants expressed a profound lack of awareness of DCD, especially when compared to other NDCs.”
We have now clarified that the lack of awareness was reported at a societal level, and that the lack of support as a result of this was evident in healthcare, education and the wider community.

K. Subtheme 2.9: Huge hole in community level support and advocacy
Please consider reframing “huge hole” which comes across as colloquial. Consider “gap” or “considerable gap” instead.
“participants felt a sense of abandonment” please consider instead “participants reported a sense…”

We thank the reviewer for both of these suggestions. "Huge hole" has been replaced with "considerable gap" throughout the manuscript, and the language regarding participants' sense of abandonment has been revised to "participants reported" in line with the more neutral, participant-centred framing applied throughout the revised manuscript.

M. Co-occurring conditions across themes
Please consider more consistently acknowledging the presence of co-occurring neurodevelopmental conditions across subthemes, rather than addressing this only in selected sections. Given the prominence of co-occurrence within the sample, a brief reminder within relevant subthemes may help avoid over-attribution of experiences solely to DCD.
We have included blurb regarding NDCs for each section of the results, where appropriate. We have also weaved this throughout the discussion where appropriate to ensure the reader has all of the information necessary to interpret the findings.

5. Discussion
A. Please consider reframing emotive language to more neutral language, eg: “belittling”.
B. Please consider reframing “alluded” throughout the transcript to “reported”, “recounted”, “identified” etc.
C. Please consider reframing “rites of passage” to “functional milestones”, “developmental stages”, etc.
We have addressed all three of these points throughout the manuscript, as described in our responses to the corresponding Results comments above. All instances of "belittling," "rites of passage" and "alluded" have been replaced with more neutral language throughout.

D. Please consider throughout the discussion section including existing qualitative research where similar themes have or have not been considered or addressed previously, and whether these themes are upheld or challenged relative to previous research, even if lifespan or childhood research. If none is available – this can also be acknowledged in the discussion section.
We have reviewed the Discussion section in its entirety and have incorporated references to existing qualitative research across all relevant sections, including one newly identified paper published after the original submission of this manuscript. Where no relevant qualitative research exists for a particular theme or subtheme, we have explicitly acknowledged this gap in the text, which we feel itself constitutes an important contribution of the current study.

E. “appeared to suffer at the hands”. Please consider using neutral language rather than emotive language.
We have revised this phrase throughout the manuscript to read "reported negative experiences due to," which reflects the participant-centred framing we have applied across the revised manuscript.

F. Causal and diagnostic implications
Please consider reviewing the discussion section holistically to ensure that language does not imply causal relationships or diagnostic utility that cannot be supported by the qualitative design. In several places, emotional experiences and functional challenges are discussed in ways that suggest DCD as a primary explanatory mechanism. Greater caution in attributing causality, and clearer framing of findings as participant-reported associations and interpretations, would strengthen the discussion.

Please reconsider this statement, a qualitative study cannot comment on causation regardless. This sentence would benefit from greater clarity and precision. The phrasing is somewhat vague and may understate an important methodological limitation. It may be helpful to more explicitly acknowledge the limits of causal interpretation inherent in the study design and to clarify where and how interpretive uncertainty arose. Please also state how future research could address this and in what way.
While this issue is acknowledged in the limitations, similar causal or attributional language appears elsewhere in the manuscript, and addressing this solely within the limitations section may not be sufficient.
We accept that a qualitative study of this nature cannot support causal claims. We have thoroughly reviewed the Discussion and revised all instances where language implied causation, ensuring that findings are consistently framed as participant-reported associations and interpretations.

G. Diagnostic relevance
Please consider tempering statements that suggest findings may directly support identification or diagnostic processes for DCD in adulthood. While the experiences described are clearly important, stronger empirical or theoretical justification would be required to support claims relating to diagnostic utility.
We have removed all statements that implied the current findings could directly inform diagnostic processes, as we agree that the qualitative design of the study does not provide a sufficient empirical basis for such claims. The revised manuscript is careful to position findings as contributing to an understanding of lived experience rather than as having direct diagnostic utility.

6. Limitations
A. co-morbid Autism – please consider using the term co-occurring.
We thank the reviewer for highlighting this terminological inconsistency. We have replaced all instances of "co-morbid" with "co-occurring" throughout the manuscript to ensure consistent use of language.

B. “Although this reflects the reality of DCD diagnoses [16] in places it was challenging to disentangle causation, resulting in an element of speculation.”
We have amended the language here to ensure clarity: *Although this reflects the reality of DCD diagnoses, in which there are often one or more co-occurring NDC diagnoses, [17], this makes it challenging to determine whether experiences were primarily attributable to DCD, co-occurring conditions, or their interaction. However, participants attributed these experiences to their DCD diagnosis, as opposed to any co-occurrence, so it is important to acknowledge these claims within the realm of lived experience.*

C. “and we were interviewing adults who may not have been able to access such historic evidence.” This is speculative as the researchers do not know whether or not the participants had access to this information.
This has now been removed from the manuscript.


D. Scope of inference
Please consider explicitly stating that the findings reflect the experiences of a small qualitative sample and may not be representative of all adults with DCD. Clarifying the scope and transferability of the findings would further strengthen this section.
We agree that this was an important clarification to include. We have now added an explicit statement at the opening of the Limitations section acknowledging that the findings reflect the experiences of a small qualitative sample of ten participants and should not be taken as representative of all adults with DCD.

7. Conclusion
A. “Individuals with DCD often feel alone, isolated and misunderstood” Please consider reframing to “often report feeling…”
We thank the reviewer for this suggestion. This phrasing has been revised to "often report feeling" in both the conclusion and the abstract, in keeping with the participant-centred and non-causational language applied throughout the revised manuscript.

B. “Data revealed that there is an urgent need for the profile of DCD to be raised” Before this statement please summarise the actual results of the data, i.e., data revealed themes and subthemes around different aspects of life, for example, … followed by the implication that these data indicate a need to raise the profile.
Unfortunately, the conclusion section requires more specificity as it is currently somewhat vague about the results and conclusions.
C. Alignment with data
Please consider ensuring that all concluding statements are clearly traceable to the themes and subthemes presented in the Results section. Where broader implications are drawn, it may be helpful to explicitly signal these as interpretations or implications rather than direct findings.
We are grateful to the reviewer for this constructive feedback. We have substantially rewritten the conclusion in response to these comments. The revised version opens by summarising the two major themes and key subthemes identified in the analysis, explicitly referencing the impact of DCD on motor skills in childhood, cognitive load in everyday activities, employment opportunities, and mental health and wellbeing across the life course, as well as the pervasive lack of societal awareness and support across education, healthcare, the family context, and the wider community. The implication that there is a need to raise the profile of DCD is then clearly positioned as arising from the cumulative weight of these findings, and language such as "there is reason to believe" is used throughout to signal where statements move beyond direct findings into broader inference.
