# Supplementary material for: ‘The world is just so fast, and I’m not fast… it’s just really, really difficult to keep up’: A qualitative exploration of the lived experience of adults with Developmental Coordination Disorder
Source: PLoS One. 2026 May 28;21(5):e0350273. doi: 10.1371/journal.pone.0350273 (PMC13218497; doi:10.1371/journal.pone.0350273)
Supplement: Attachment — Submitted filename: Response to reviewers (1).docx [file pone.0350273.s006.docx]

Reviewer #1: This manuscript addresses an important and underexplored area, offering valuable insight into the lived experiences of adults with DCD. The qualitative data presented are rich and meaningful, and the paper makes a worthwhile contribution to the literature. While the manuscript is generally well-structured and clearly written, a small number of revisions would strengthen clarity and impact. Only a few minor queries remain outstanding, and I would recommend publication once these have been addressed.

We thank the reviewer for their kind words, and for their support in getting the manuscript to this point!

Introduction
The statement that tasks such as cooking and cleaning leave adults with DCD feeling “tired” [12] may not fully convey the extent of fatigue and overwhelm described in the literature and by lived experience. Consider strengthening this wording to better reflect the intensity and cumulative burden of these everyday activities for adults with DCD.

Thank you for the suggestion! This had been toned down based on previous reviewer feedback, however, we agree that the previous wording does not fully capture experiences of those with DCD so we have changed this to: *‘enduring the cumulative burden of consequential fatigue which plays a significant role in their day to day lives’*.

Results
Some sub-themes include a substantial amount of dialogue-based evidence, which is rich and insightful. However, this section would benefit from greater concision, as its current length may risk reducing reader engagement. Streamlining the number of quotations; selecting two to three representative examples per sub-theme would enhance clarity and reinforce the key messages without diminishing the depth of analysis.

We thank the reviewer for highlighting the length of the manuscript may impact reader engagement. As a response, we have been through the quotes in the manuscript and reduced all sections to a maximum of 3 quotes.

Reviewer #2: Overall Recommendation: Minor Revision

The authors have engaged constructively with prior feedback and the manuscript has improved substantially. The study makes a valuable contribution to the qualitative literature on adults with DCD.

We thank the reviewer acknowledging the work undertaken to the manuscript to get to this point. We really appreciate their support thus far.

One minor point remains regarding the Discussion: in a few places, participant-reported experiences are still framed in ways that extend toward broader societal-level conclusions (e.g., inequity, systemic lack of support). While these interpretations are plausible, slightly more cautious wording would better align with the qualitative scope of the data.

We thank the reviewer for this observation. We recognise that in places the Discussion extended beyond the scope of the qualitative data by framing participant experiences as broader societal-level conclusions. We have revisited the Discussion and adjusted the language throughout to ensure that claims are appropriately anchored to participants' accounts. For example, 'collectively revealed a society which is failing to scaffold vulnerable adults' has been revised to foreground participant voice, and phrases such as 'inequitable support' and 'reveals the need' have been reframed as participant perceptions rather than stated as objective fact. We believe these changes better reflect the qualitative scope of the data whilst preserving the substance of the findings.
